# Clinical concentration of sevoflurane had no short-term effect on the myelin sheath in prefrontal cortex of aged marmosets

**DOI:** 10.3389/fnins.2024.1447743

**Published:** 2024-08-08

**Authors:** Zhengjie Miao, Yi Jiang, Fangfang Wang, Lingling Shi, Ren Zhou, Yixuan Niu, Lei Zhang

**Affiliations:** ^1^Department of Anesthesiology, Shanghai Ninth People’s Hospital, Shanghai Jiao Tong University School of Medicine, Shanghai, China; ^2^Shanghai Institute of Precision Medicine, Ninth People’s Hospital, Shanghai Jiao Tong University School of Medicine, Shanghai, China

**Keywords:** general anesthetic, primate, myelin sheath, scanning electron microscopy, sevoflurane

## Abstract

**Introduction:**

The fragile brain includes both the developing brain in childhood and the deteriorating brain in elderly. While the effects of general anesthesia on the myelin sheath of developing brain have been well-documented, limited research has explored its impact on degenerating brain in elderly individuals.

**Methods:**

In our study, aged marmosets in control group were only anesthetized with 6–8% sevoflurane and 100% oxygen (2 L/min) for 1–2 min for anesthesia induction. In addition to anesthesia induction, the anesthesia group was exposed to a clinical concentration of sevoflurane (1.5–2%) for 6 h to maintain anesthesia. After anesthesia, scanning electron microscopy (SEM) and artificial intelligence-assisted image analysis were utilized to observe the effects of general anesthesia on the myelin sheath in prefrontal cortex (PFC) of aged marmosets.

**Results:**

Compared with the control group, our findings revealed no evidence that 6 h of sevoflurane general anesthesia altered the thickness of myelin sheath, the diameter of myelinated axons, and the *g*-ratio in prefrontal cortex of aged marmosets.

**Conclusion:**

Clinical concentration of sevoflurane may have no short-term effect on the myelin sheath in prefrontal cortex of aged marmosets.

## Introduction

With the advancement of surgery, anesthesia technology and the aging of global population, the number of elderly patients undergoing surgical procedures is increasing. General anesthetics act directly on the brain, making the aged brain particularly vulnerable to injury during surgery, which surgery is a form of trauma. This dual impact puts the cognitive function of elderly patients at high risk during the perioperative period, potentially leading to perioperative neurocognitive disorder (PND). PND results in increased hospitalization costs, longer hospital stays, higher postoperative mortality rates, a serious impact on the physical and mental health of elderly patients. Furthermore, it creates a significant financial strain on both society and the families of those affected. The impact of general anesthetics on the aged brain has therefore become a significant topic in perioperative medicine.

The maintenance of myelin integrity is associated with the functions of the central nervous system (CNS), which involve cognitive, learning, and memory processes. The disruption of myelin structure can result in impaired motor function and cognitive deterioration (Franklin and Ffrench-Constant, 2017; Gibson et al., 2019; McNamara et al., 2023). While previous research has indicated that general anesthesia can harm the myelin sheath of the developing brain during childhood (Li et al., 2019; Zhang et al., 2019; Wu et al., 2020; Zuo et al., 2020), the impact of general anesthetics on myelin in the aged brain has largely been unexplored. In our earlier research, it was discovered that general anesthesia does not increase the levels of neuroinflammation factors; however, it does enhance glycolysis and lactate production in the brain of aged marmosets (Cheng et al., 2022; Zhang et al., 2022). Studies have indicated that glycolysis is intricately linked to the maintenance of myelin integrity (Fünfschilling et al., 2012). Previous research also indicated that persistently increased neuronal metabolism of lactate causes oxidative stress, leading to impairment in neuroenergetics and axon stability, including reduced myelin thickness of the nerves (Jia et al., 2021). Based on our research and related reports, we further evaluate the impact of sevoflurane anesthesia on myelin in aged brain in the research.

Since patients only receive general anesthesia during surgery, and it is challenging to distinguish the effects of general anesthesia from those of surgery on the brain, the study utilizes aged marmosets as experimental subjects. Using non-human primates in brain research offers significant advantages due to their close physiological and anatomical similarity to humans. Their complex cognitive abilities and behaviors, coupled with their genetic proximity to humans, make them ideal models for studying advanced brain functions and neurobehavioral diseases. This approach, while ethically regulated, allows for deeper and more controlled experimental studies than direct human research.

The objective of this study was to investigate the short-term effects of general anesthesia on the myelin sheath in the prefrontal cortex (PFC) of aged marmosets. We hypothesized that exposure to sevoflurane during general anesthesia might lead to alterations in the myelin sheath, including changes in its thickness and the *g*-ratio in the PFC. The results of this study could provide valuable insights into the mechanisms behind postoperative cognitive dysfunction resulting from general anesthesia in elderly individuals.

## Materials and methods

### Marmoset anesthesia

The marmoset research adhered to the guidelines and regulations set by the Institute of Animal Care Committee at the Center for Excellence in Brain Science and Intelligence Technology (CEBSIT), Chinese Academy of Sciences [License No. SYXK (Shanghai) 2021-0003]. The study received approval from the Institutional Animal Care and Use Committee of the Institute of Experimental Animal Science (Protocol No. CEBSIT-2021035). To minimize the number of animals used, four marmosets (*Callithrix jacchus*, 4 males, weighing 234–286 g) aged over 8 years were individually housed in a facility with controlled temperature (25–27°C) and humidity (30–70%). They were kept on a 12 h light/dark cycle and provided with water and a balanced diet *ad libitum*.

Two marmosets (2 males) were assigned to the sevoflurane anesthesia group. These marmosets received 6–8% sevoflurane and 100% oxygen (2 L/min) for anesthesia induction (1–2 min), followed by 1.5–2.5% sevoflurane and 100% oxygen (2 L/min) for maintaining anesthesia. Sevoflurane was administered through mask ventilation without intubation (Coleman et al., 2017; Zhang et al., 2022). Another two marmosets (2 males) were included in the control group and were anesthetized with 6–8% sevoflurane and 100% oxygen (2 L/min) for anesthesia induction for 1–2 min. Vital signs were monitored using BeneVision M12 monitors (Mindray, China) during general anesthesia, and a warm blanket (AHM06, Reptizoo, China) was used to maintain a temperature of 37 ± 0.5°C. At the end of sevoflurane anesthesia, the marmosets were humanely euthanized under deep anesthesia with 3–5% sevoflurane. Prior to euthanasia, blood was drawn from the femoral artery for blood gas analysis using a portable clinical analyzer (i-STAT; Abbott Laboratories Inc., East Windsor, NJ, United States). The characteristics and results of the blood gas analysis are shown in Supplementary Table S1. We maintained the marmosets’ body temperature at 37°C using an animal warming mat system (AHM06, Reptizoo, China) and monitored their heart rate, electrocardiogram, respiratory rate, peripheral capillary oxygen saturation (SpO_2_), and rectal temperature using patient monitors (BeneVision M12, Mindray, China). After administering anesthesia, we performed a cardiac puncture and used a portable clinical analyzer (i-STAT; Abbott Laboratories, East Windsor, New Jersey, United States) to analyze arterial blood gas. To prevent dehydration, the marmosets were infused with 5 mL of stroke-physiological saline solution every 2 h.

### Sample preparation and scanning electron microscopy imaging

**Figure 1:**
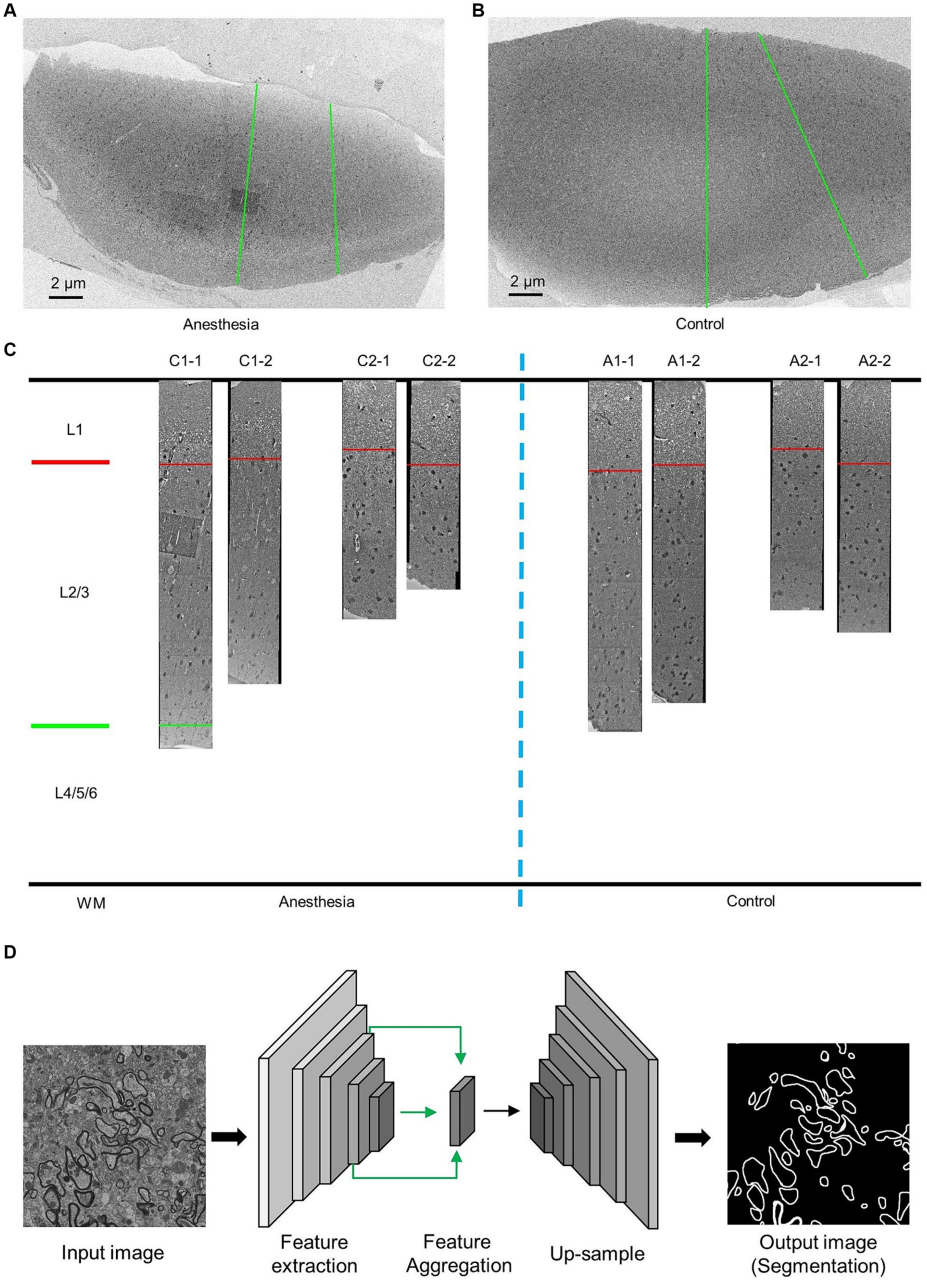
EM data description and AI-assisted analysis of the prefrontal cortex in the aged marmosets. **(A,B)** Low-resolution images of the prefrontal cortex slices. The green line indicates the direction of high-resolution imaging. **(C)** The EM data and layering of the prefrontal cortex slices. **(D)** AI-assisted myelin segmentation model. The input of the model is an EM image, and the output is the mask of the myelin. The scale bar is 2 μm in **A,B**.

After euthanizing the marmosets, we perfused their brain tissue with phosphate-buffered saline (PBS) to collect prefrontal cortex samples for SEM observation. The tissue was first fixed with 2.5% glutaraldehyde, followed by 1% osmium peroxide, and then stained with uranyl acetate and lead citrate. After dehydration, the tissue was embedded in silicone resin. Imaging was performed using a Zeiss scanning electron microscope (GeminiSEM 300) at a working voltage of 5 kV and a resolution of 12 nm/pixel, with each slice being imaged in two columns along the surface of the prefrontal cortex toward the white matter (Figures 1A,B).

### Artificial intelligence-assisted image analysis

Images of the prefrontal cortex of marmosets were obtained using an electron microscope with a resolution of 12 nm × 12 nm (Figure 1C). Previous studies have demonstrated that the prefrontal cortex can be divided into six layers, with the first layer positioned below the skull and the sixth layer nearest to the white matter. These layers exhibit variations in cell types and functions (Burman and Rosa, 2009). Actual measurements of the inner prefrontal cortex in marmosets and the outer prefrontal cortex layers in humans show that the combined length of the second and third layers is three times greater than that of the first layer (Sasaki et al., 2015; Maynard et al., 2021). The prefrontal cortex samples we obtained were stratified accordingly (Figure 1C).

The myelin sheath in the prefrontal cortex retains myelin phospholipids when fixed and stained with osmic acid, making it easily distinguishable from nonmyelin structures via electron microscopy. Due to the dense distribution and structural complexity of the cortex, artificial intelligence technology is employed for the quantitative analysis of the myelin sheath structure.

#### Myelin sheath segmentation

We utilized deep convolutional neural networks (DCNNs) to train a model for myelin segmentation. This model is capable of categorizing each pixel in the input image as either “myelin sheaths” or “nonmyelin sheaths,” resulting in a myelin sheath segmentation image of the same scale as the input. The details of the methodology can be found in the previous study (Jiang et al., 2022) (Figure 1D).

#### Myelin sheath thickness measurement

**Figure 2:**
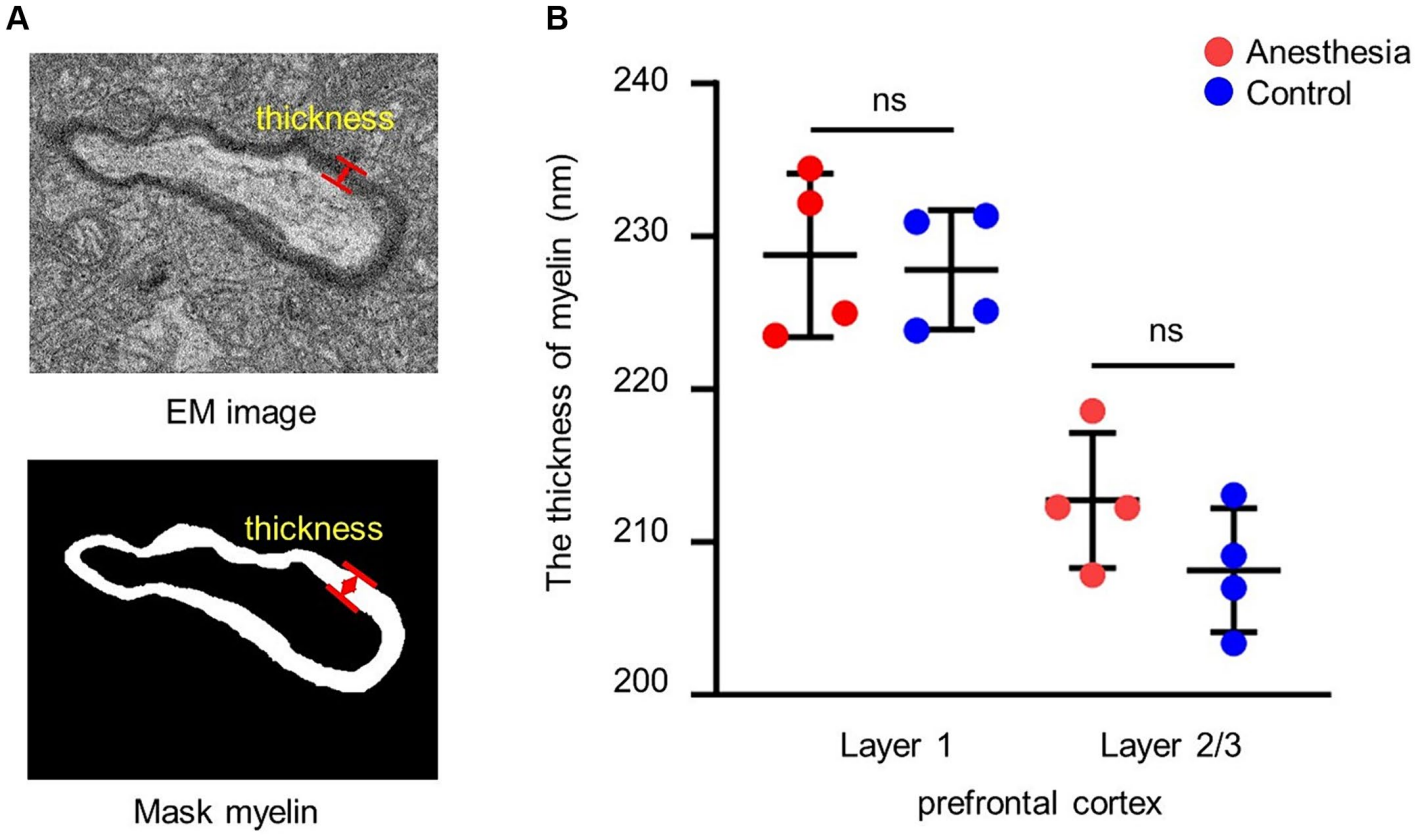
Analysis of the thickness of myelin. **(A)** EM image and mask of the myelin. The red arrow indicates the thickness of the myelin. **(B)** Statistical analysis of the myelin thickness in the brain slices of anesthesia group and control group (layer 1, *p* = 0.779, unpaired *t*-test, mean ± SD; layer 2/3, *p* = 0.177, unpaired *t*-test, mean ± SD).

To determine the average thickness of the myelin sheath, the thickness was measured at 10 positions on each sheath (Figure 2A).

#### *G*-ratio calculation

**Figure 3:**
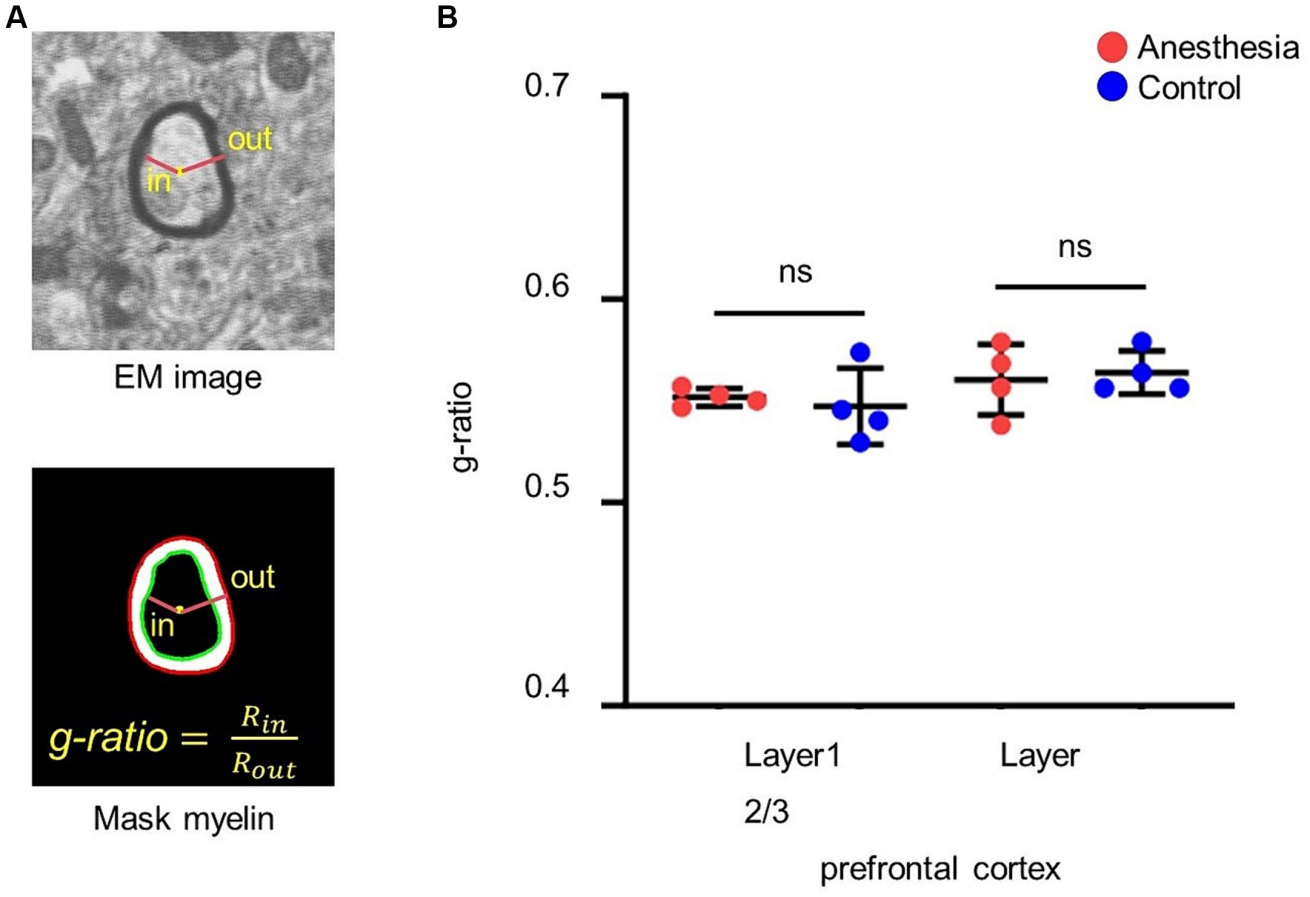
Analysis of the *g*-ratio. **(A)** *G*-ratio of myelin. *G*-ratio is the ratio of the inner diameter to the outer diameter of the myelin, and 0 < *G*-ratio < 1. **(B)** Statistical analysis of the *g*-ratio in the brain slices of anesthesia group and control group (layer 1, *p* = 0.665, unpaired *t*-test, mean ± SD; layer 2/3, *p* = 0.746, unpaired *t*-test, mean ± SD).

The center of the myelin sheath was represented by the fitted ellipse of its inner border. The ratio of the inner diameter to the outer diameter was subsequently determined through the center point (Figure 3A).

#### Diameter measurement of myelinated axons

**Figure 4:**
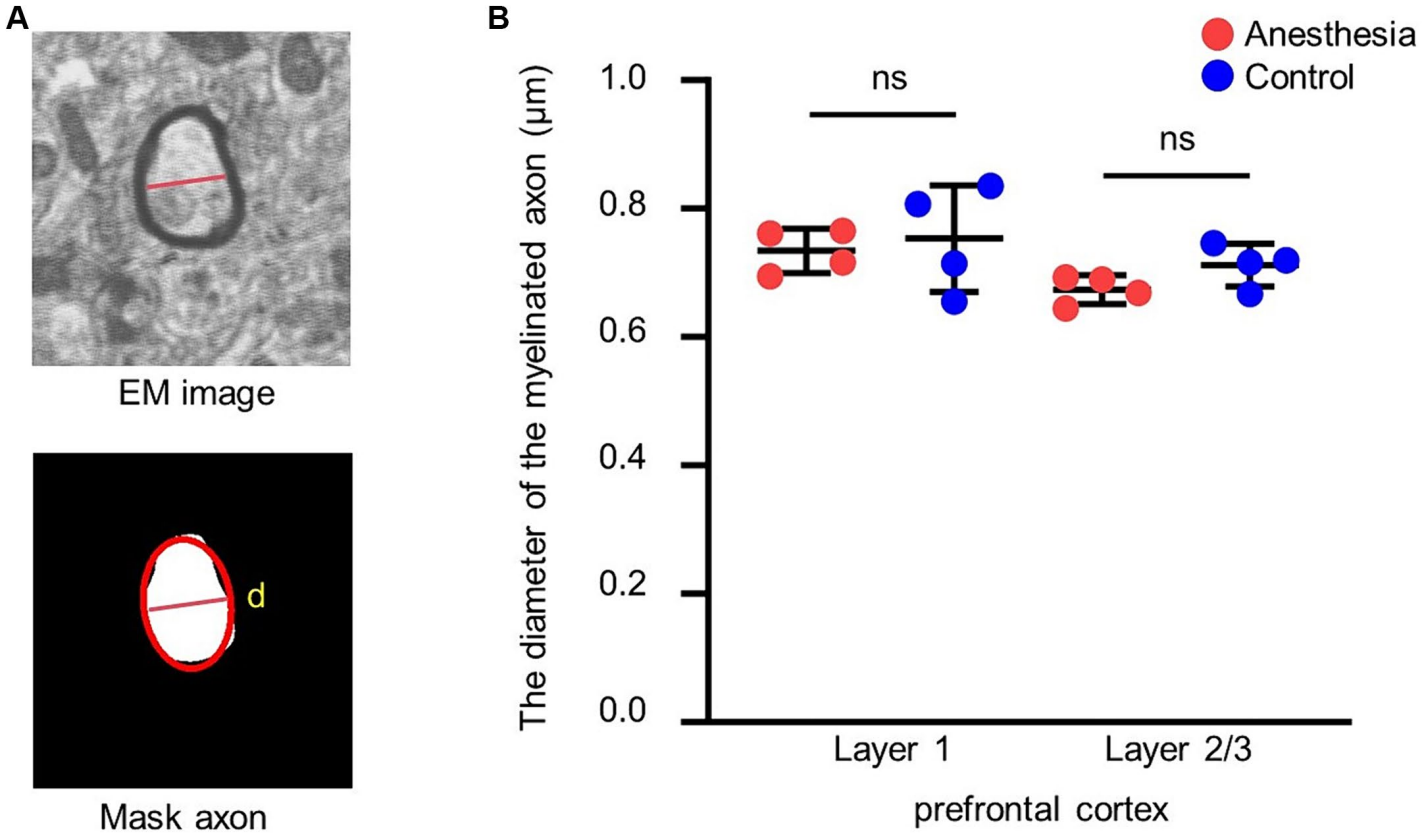
Analysis of the diameter of the myelinated axon. **(A)** EM image and mask of the myelinated axon. The red ellipse is an ellipse fit for the mask, and the line is the short axis. **(B)** Statistical analysis of the myelinated axon diameter in the brain slices of anesthesia group and control group (layer 1, *p* = 0.688, unpaired *t*-test, mean ± SD; layer 2/3, *p* = 0.105, unpaired *t*-test, mean ± SD).

Myelinated axons were subjected to elliptical fitting, where the length of the short axis of the ellipse was used to measure the diameter of the axons (Figure 4A).

### Statistical analysis

All the data analyses and statistical tests were performed using custom-written Python scripts and built-in functions (Python 3.9). We validated that each data set follows a normal distribution using the Shapiro–Wilk test. We used unpaired *t*-tests to compare the average values of the control group and the anesthesia group data. The data were presented as means ± SD. The significance was fixed at *p* < 0.05.

## Results

Using SEM and artificial intelligence technology, we analyzed the effects of sevoflurane general anesthesia on the myelin sheath of the prefrontal cortex in aged marmosets. This analysis focused on the thickness of the myelin sheath, the *g*-ratio, and the diameter of myelinated axons.

### Clinical concentration of sevoflurane had no short-term effect on myelin thickness in prefrontal cortex of aged marmosets

We examined the impact of the anesthetic on the myelin sheath of the prefrontal cortex in aged marmosets after 6 h of general anesthesia with a clinical concentration of sevoflurane. The results indicated that there was no difference in myelin sheath thickness between the control group and the anesthesia group in layer 1 (*p* = 0.779) and layer 2/3 (*p* = 0.177) of the prefrontal cortex (Figure 2B).

### Clinical concentration of sevoflurane had no short-term effect on *g*-ratio in prefrontal cortex of aged marmosets

We calculated and statistically analyzed the *g*-ratio in the prefrontal cortex of aged marmosets to investigate whether 6 h of clinical sevoflurane general anesthesia caused changes in the myelin sheath. The results indicated that there was no difference in the *g*-ratio in layer 1 (*p* = 0. 0.665) or layer 2/3 (*p* = 0.746) of the prefrontal cortex between the control group and the anesthesia group (Figure 3B).

### Clinical concentration of sevoflurane had no short-term effect on diameter of myelinated axons in prefrontal cortex of aged marmosets

We assessed whether 6 h of clinical-concentration sevoflurane general anesthesia would alter the diameter of myelinated axons in the prefrontal cortex of aged marmosets. The results indicated that there was no difference in the diameter of myelinated axons in layer 1 (*p* = 0.688) or layer 2/3 (*p* = 0.105) of the prefrontal cortex between the control group and the anesthesia group (Figure 4B).

## Discussion

In the study, aged marmosets were used as experimental animals, and sevoflurane was used for general anesthesia. Scanning electron microscopy (SEM) was employed to image brain samples from the marmosets, and artificial intelligence technology was utilized to process and analyze the images. We systematically evaluated the impact of sevoflurane anesthesia on the structure of the myelin sheath in prefrontal cortex (PFC) of aged marmosets. The results revealed that Clinical concentration of sevoflurane had no short-term effect on the thickness of the myelin sheath, the *g*-ratio and the diameter of myelinated axons of the PFC in aged marmosets.

Our previous study revealed that the myelin sheaths in the PFC of aged marmosets experienced degenerative changes. Changes in the composition of myelin basic protein (MBP) indicate the extent of demyelination, but there was no difference in MBP expression between the control group and sevoflurane group (Cheng et al., 2022). However, a thorough examination of the myelin sheath structure was not performed. In the present study, we investigated the thickness and *g*-ratio of the myelin in the prefrontal cortex of aged marmosets using high-throughput measurements. The *g*-ratio has been reported to remain relatively consistent in the white matter of both healthy non-human primate brains and human brains, with values approaching 0.7. In the multiple sclerosis brain, the *g*-ratio was elevated in lesions. *G*-ratio values above 0.8 are indicative of acute demyelination (Stikov et al., 2015). The study showed that the *g*-ratio in the prefrontal cortex of aged marmosets after sevoflurane anesthesia is approaching 0.7 and has no difference with the control group, indicating that sevoflurane general anesthesia did not cause myelin sheath damage in the brain of elderly marmosets at the early stages of anesthesia.

The potential cause of the results may be that the neurotoxicity of sevoflurane significantly affects myelination during neurodevelopment, rather than during the aging process. During the initial stages of brain development, nerve cells are primarily involved in proliferation and growth, essential for maintaining normal brain function. Apoptosis is not commonly observed under normal conditions. However, under abnormal conditions, nerve cells may undergo apoptosis due to cellular stress or drug exposure, and this change is readily observable. In elderly individuals, damage to the myelin sheath in the PFC may already be present. In humans, the most significant age-related reductions in white matter integrity occur in anterior brain regions, which have been linked to declines in processing speed and executive functioning (Madden et al., 2012). In aged rats, the myelin sheath shows increasing fragmentation and detachment from the axon, a phenomenon linked to reductions in structural proteins like myelin basic protein (MBP) and cyclic nucleotide phosphodiesterase (Sugiyama et al., 2002; Liu et al., 2017). Though the effects of sevoflurane anesthesia can worsen the damage, it may not produce significant differences. Age disparity could be a contributing factor to the varying effects of sevoflurane anesthesia on the developing brain in childhood versus the degenerating brain in elderly individuals, warranting further investigation and confirmation. It is also possible that the damage to myelin in the aged brain may not manifest at the early stage of anesthesia, as our previous research has shown that anesthesia affects lactate metabolism in aged marmosets (Zhang et al., 2022), and persistently increased neuronal metabolism of lactate reduced myelin thickness of the nerves (Jia et al., 2021). Future studies are needed to investigate the long-term effects of sevoflurane anesthesia on myelin in the aged brain.

There are several limitations to our study. First, we harvested brain tissue immediately following sevoflurane anesthesia, which may not have allowed sufficient time for any myelination deficits to become apparent. This study specifically focused on the acute effects of sevoflurane exposure. To determine the long-term effects, the sampling period should be extended appropriately. Second, due to the precious and scarce nature of nonhuman primate experimental animals, the sample size for this study was limited.

We have implemented measures to compensate for the limitation of sample size. These images were processed using deep convolutional neural networks (DCNNs) trained on a myelin segmentation model. The use of DCNNs for training medical image segmentation models has reached a high level of maturity. Prior research has utilized DCNN technology to achieve precise segmentation through electron microscopy imaging of the ventral neural cord (VNC) of fruit fly larvae (Ronneberger et al., 2015). Additionally, we have effectively utilized artificial intelligence technology to aid in the analysis of medical images, leading to the identification of a decrease in synapses in the prefrontal cortex due to Alzheimer’s disease (Jiang et al., 2022). In the present study, we continued to employ this technology to address the challenges of identifying, counting, and measuring various myelin sheath parameters in images. In the future, the results need to be further validated using aged rodent models with a larger sample size.

## Data availability statement

The raw data supporting the conclusions of this article will be made available by the authors, without undue reservation.

## Ethics statement

The animal study was approved by the Animal Care Committee of the Center for Excellence in Brain Science and Intelligence Technology (CEBSIT, China) and the Institutional Animal Care and Use Committee (Protocol Number CEBSIT-2021035). The study was conducted in accordance with the local legislation and institutional requirements.

## Author contributions

ZM: Conceptualization, Investigation, Writing – original draft. YJ: Formal analysis, Methodology, Software, Visualization, Writing – original draft. FW: Methodology, Visualization, Writing – original draft. LS: Data curation, Writing – original draft. RZ: Data curation, Writing – original draft. YN: Data curation, Writing – original draft. LZ: Conceptualization, Project administration, Resources, Supervision, Writing – review & editing.
