# Supplementary material for: Clinical concentration of sevoflurane had no short-term effect on the myelin sheath in prefrontal cortex of aged marmosets
Source: Front Neurosci. 2024 Aug 8;18:1447743. doi: 10.3389/fnins.2024.1447743 (PMC11338887; doi:10.3389/fnins.2024.1447743)
Supplement: Supplementary file 1 [file Table_1.DOCX]

**Supplementary Table S1. The characteristics of the aged marmosets between the two groups**

Continuous variables are median (standard error of the mean). HR, heart rate; RR, respiratory rate; T, body temperature; SPO2, transcutaneous oxygen saturation; pH, Arterial pH; PaO2, arterial partial pressure of oxygen; PaCO2, arterial partial pressure of carbon dioxide; BE (ecf), arterial blood actual alkali reserve or alkali surplus; TCO2, Total carbon dioxide; SO2, oxygen saturation.

|  | **Control group**  **(n=2)** | **sevoflurane anesthesia group**  **(n=2)** | ***P* value** |
| --- | --- | --- | --- |
| Age(yr) | 8.50 (0.50) | 8.50 (0.50) | >0.999 |
| Sex | 2M | 2M |  |
| Weight (g) | 247.50 (13.50) | 285.00 (1.00) | 0.109 |
| HR (bmp min^-1^) | 183.00 (4.00) | 191.50 (1.50) | 0.185 |
| RR (r min^-1^) | 43.50 (3.50) | 46.00 (3.00) | 0.642 |
| T (℃) | 37.50 (0.20) | 36.85 (0.25) | 0.179 |
| SPO2 (%) | 99.50 (0.50) | 99.00 (1.00) | 0.699 |
| pH | 7.39 (0.02) | 7.62 (0.06) | 0.061 |
| PaO2 (kPa) | 41.76 (25.00) | 33.32 (0.33) | 0.768 |
| PaCO2 (kPa) | 6.28 (1.35) | 4.89 (1.01) | 0.497 |
| BE (ecf) (mmol L^-1^) | 3.00 (5.00) | 15.50 (1.50) | 0.139 |
| HCO3^-^ (mmol L^-1^) | 28.10 (5.00) | 36.75 (2.85) | 0.272 |
| TCO2 (mmol L^-1^) | 29.50 (5.50) | 38.00 (3.00) | 0.308 |
| SO2 (%) | 99.50 (0.50) | 100.00 (0.00) | 0.423 |
